# Baseline vs. cross-sectional MRI of concussion: distinct brain patterns in white matter and cerebral blood flow

**DOI:** 10.1038/s41598-020-58073-9

**Published:** 2020-02-03

**Authors:** Nathan W. Churchill, Michael G. Hutchison, Simon J. Graham, Tom A. Schweizer

**Affiliations:** 1 Neuroscience Research Program, St. Michael’s Hospital, Toronto ON, M5B 1M8 Canada; 2 Keenan Research Centre for Biomedical Science of St. Michael’s Hospital, Toronto ON, M5B 1M8 Canada; 3 Faculty of Kinesiology and Physical Education, University of Toronto, Toronto ON, M5S 2C9 Canada; 4 Department of Medical Biophysics, University of Toronto, Toronto ON, M5G 1L7 Canada; 5 Sunnybrook Research Institute, Sunnybrook Hospital, Toronto ON, M4N 3M5 Canada; 6 Faculty of Medicine (Neurosurgery), University of Toronto, Toronto ON, M5T 1P5 Canada

**Keywords:** Neuroscience, Brain injuries

## Abstract

Neuroimaging has been used to describe the pathophysiology of sport-related concussion during early injury, with effects that may persist beyond medical clearance to return-to-play (RTP). However, studies are typically cross-sectional, comparing groups of concussed and uninjured athletes. It is important to determine whether these findings are consistent with longitudinal change at the individual level, relative to their own pre-injury baseline. A cohort of N = 123 university-level athletes were scanned with magnetic resonance imaging (MRI). Of this group, N = 12 acquired a concussion and were re-scanned at early symptomatic injury and at RTP. A sub-group of N = 44 uninjured athletes were also re-imaged, providing a normative reference group. Among concussed athletes, abnormalities were identified for white matter fractional anisotropy and mean diffusivity, along with grey matter cerebral blood flow, using both cross-sectional (CS) and longitudinal (LNG) approaches. The spatial patterns of abnormality for CS and LNG were distinct, with median fractional overlap below 0.10 and significant differences in the percentage of abnormal voxels. However, the analysis methods did not differ in the amount of change from symptomatic injury to RTP and in the direction of observed abnormalities. These results highlight the impact of using pre-injury baseline data when evaluating concussion-related brain abnormalities at the individual level.

## Introduction

Concussion in sport and recreation is a significant health concern, with an estimated 3.8 million occurring every year in North America^1^ and growing evidence of potential long-term health consequences^2^. At present, symptom status, brief cognitive screening, and balance assessments form the cornerstones of concussion management in sport and recreation^3^. Diagnosis is mainly based on an observed mechanism of injury and behavioural manifestations; safe return to play (RTP) is subsequently determined based on symptom resolution and the completion of a graded exercise protocol^3^. Although the clinical features of concussion have been well-characterized^4–7^, these assessments only indirectly reflect the underlying brain injury and recovery process, which remain incompletely understood in humans.

Advanced magnetic resonance imaging (MRI) has been used to describe the pathophysiology of concussion and to determine whether recovery persists beyond medical clearance to RTP^8,9^. Diffusion tensor imaging (DTI) has been widely used to assess altered white matter microstructure among concussed athletes^10–14^. In addition, arterial spin labelling (ASL) has been increasingly used to evaluate changes in cerebral blood flow which occur after a concussion, particularly during the early symptomatic phase of injury^14–17^. Despite the rapid pace of research, however, consensus guidelines do not yet consider these tools to be sufficiently mature to inform clinical practise^3^.

One barrier to clinical relevance is the limited amount of prospective, subject-specific neuroimaging data, with the majority of MRI literature comparing groups of concussed and uninjured athletes^9^. Our present understanding of concussion pathophysiology, and whether the brain is recovered at RTP, is therefore mainly based on the assumptions that (1) the uninjured group is representative of the pre-injury brains of concussed athletes and (2) group means provide an effective summary of concussion effects at the individual level. However, MRI studies have reported significant baseline variations in brain physiology between individuals^17–20,^ and both neuroimaging^21^ and clinical studies^22^ have reported significant inter-individual variability in acute presentation and the timeline of recovery. Therefore, it is imperative to also investigate the effects of concussion at the individual level, relative to athletes’ own pre-injury brains. While there is a growing literature base examining concussion effects at the individual level^23,24^, including emerging results with pre-injury baseline data^25–27^, this remains an under-studied area of research. It is as of yet unclear whether concussion-related abnormalities identified in longitudinal analysis relative to baseline imaging are distinct from those identified in cross-sectional comparisons to uninjured cohorts.

The present study investigated this issue, by imaging a large cohort of athletes at the start of their athletic season, using DTI and ASL. Athletes that had sustained a concussion during sport participation were re-imaged, both at the early symptomatic phase of injury (SYM) and at RTP. As a normative reference, a group of athletic controls (i.e., athletes that did not sustain a concussion) were also re-scanned at the end of their competitive season. The study compared brain abnormalities detected at the individual subject level using a cross-sectional analysis approach (i.e., comparing the brains of concussed athletes, relative to brains of athletic controls) and using a longitudinal analysis approach (i.e., comparing the brain changes of concussed athletes from baseline to post-injury, relative to longitudinal changes of athletic controls). It was hypothesized that longitudinal analyses would detect concussion-related brain abnormalities that are distinct from cross-sectional analyses, both at SYM and RTP.

## Results

### Demographics and clinical data

A total of 123 athletes were imaged prior to the start of their competitive season. From this group, 12 athletes (10% of the cohort) sustained a concussion over the course of the study (median and [Q1, Q3]: 85 [15, 217] days from baseline imaging). They were re-imaged at both SYM (4 [3, 6] days post-concussion) and RTP (38 [17, 97] days post-concussion), with N = 10 for DTI and N = 11 for ASL (symptomatic) and N = 11 for DTI and N = 12 for ASL (RTP). Athlete symptoms were assessed using the sport concussion assessment tool (SCAT), at baseline and both post-concussion timepoints. In addition, a group of 44 athletic controls, drawn from the remaining athletes that did not sustain a concussion, were re-imaged at the end of their athletic season. This group provided normative data for identifying brain abnormalities of concussed athletes, both cross-sectionally and longitudinally.

Table 1 summarizes the cohort demographics. The concussed athletes had elevated SCAT symptom severity and total symptoms while symptomatic, relative to their baseline (*p* = 0.008 and *p* = 0.013 respectively, non-parametric paired Wilcoxon tests), whereas at RTP, symptom severity and total symptoms were no longer elevated relative to their baseline (*p* = 0.490 and *p* = 0.474, respectively). Baseline symptom severity for the N = 12 concussed athletes was also shown to be comparable to the athletic control group (*p* = 0.452 and *p* = 0.490, 2-sample Wilcoxon tests). For the concussed athletes at baseline, 4 had prior concussion (N = 2 had one previous concussion and N = 2 had 2 previous concussions; all were sustained between 12 and 49 months prior to baseline imaging). Table 2 summarizes athlete numbers by sport for the complete athletic cohort, the normative subset and the concussed athletes. All groups consisted of a mixture of different sport types, including non-contact, limited contact and collision^28^.

**Table 1:** Participant demographics. Age is reported as mean ± standard deviation. Clinical statistics are based on the Sport Concussion Assessment Tool (SCAT) and reported as median and [Q1, Q3]. Results are reported for a total of N = 12 subjects, however scanning N = 10 for DTI and N = 11 for ASL (symptomatic) and N = 11 for DTI and N = 12 for ASL (RTP).

	All baselines	Control group	Concussed		
			Baseline	Symptomatic	RTP
Age	20.3 + −2.0	20.0 + −1.8	20.3 + −1.5	—	—
Female	61/123 (50%)	24/44 (55%)	5/12 (42%)	—	—
previous concussion	57/123 (46%)	18/44 (31%)	4/12 (33%)	—	—
SCAT total symptoms	2 [0 5]	2 [1 4]	2 [1 7]	5 [4 14]	0 [0 2]
SCAT symptom severity	2 [0 7]	2 [1 6]	2 [1 17]	5 [5 35]	0 [0 3]

**Table 2:** Athlete numbers by sport, for both male (M) and female (F) groups.

All baselines	Control group	Concussed
Volleyball (13 M, 12 F)	Volleyball (3 M, 1 F)	Volleyball (1 F)
Ice hockey (17 M)	Ice hockey (6 M)	Ice hockey (4 M)
Field hockey (24 F)	Field hockey (13 F)	Field hockey (1 F)
Soccer (10 M, 7 F)	Soccer (2 M, 3 F)	Football (2 M)
Football (9 M)	Football (4 M)	Rugby (1 M, 2 F)
Rugby (3 M, 9 F)	Rugby (1 M, 1 F)	Basketball (1 F)
Basketball (7 F)	Basketball (4 F)	
Lacrosse (9 M, 2 F)	Lacrosse (4 M, 2 F)	
Water polo (1 M)		

### Neuroimaging data: normative group

**Figure 1:**
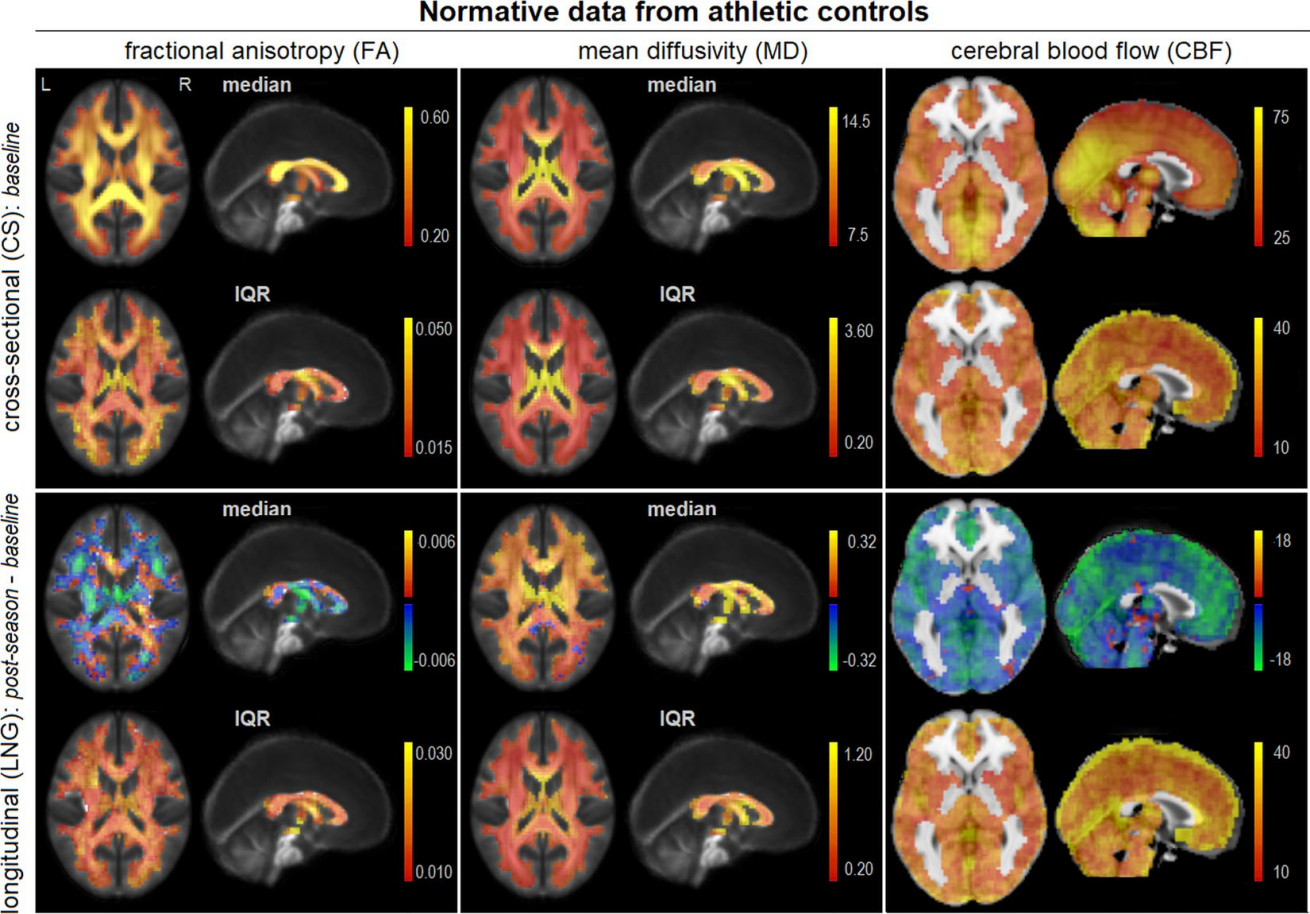
Normative data from the 44 athletic controls used to perform cross-sectional (CS) and longitudinal (LNG) analyses, including voxel-wise median and interquartile range (IQR). The CS maps are from baseline MRI parameter values and LNG maps are from the longitudinal changes in MRI parameter values from pre-season to post-season. Representative slices are shown for DTI data (x = + 0, z = + 18) and ASL data (x = + 0, z = + 0). The plots show expected normative ranges of variability for all MRI parameters.

For all athletes, DTI was used to measure the fractional anisotropy (FA) and mean diffusivity (MD) within white matter tracts and ASL was used to quantify cerebral blood flow (CBF) of grey matter tissue. For the group of 44 athletic controls, Fig. 1 plots distribution statistics for cross-sectional (CS) pre-season baseline data, as well as the longitudinal (LNG) changes from baseline to post-season. For athletic control CS data, the coefficient of variation was measured over baseline scan values at each voxel, and summarized by the median and [Q1, Q3] over voxels, for FA (0.0586, [0.0468, 0.0766]), MD (0.0423, [0.0324, 0.0664]) and CBF (0.443, [0.368, 0.562]). For athletic control LNG data, coefficients of variation over longitudinal change values were substantially higher, for FA (7.47, [4.37, 15.81]), MD (2.26, [1.63, 3.88]) and CBF (2.99, [2.27, 4.27]). The longitudinal median percent signal change relative to baseline was also measured at each voxel and was summarized by the median and [Q1, Q3] over voxels, for FA (−0.12%, [−0.55%, 0.33%]), MD (1.41%, [0.76%, 2.02%]) and CBF (−1.87% [−2.55%, −1.32%]).

### Neuroimaging data: concussed athletes

**Figure 2:**
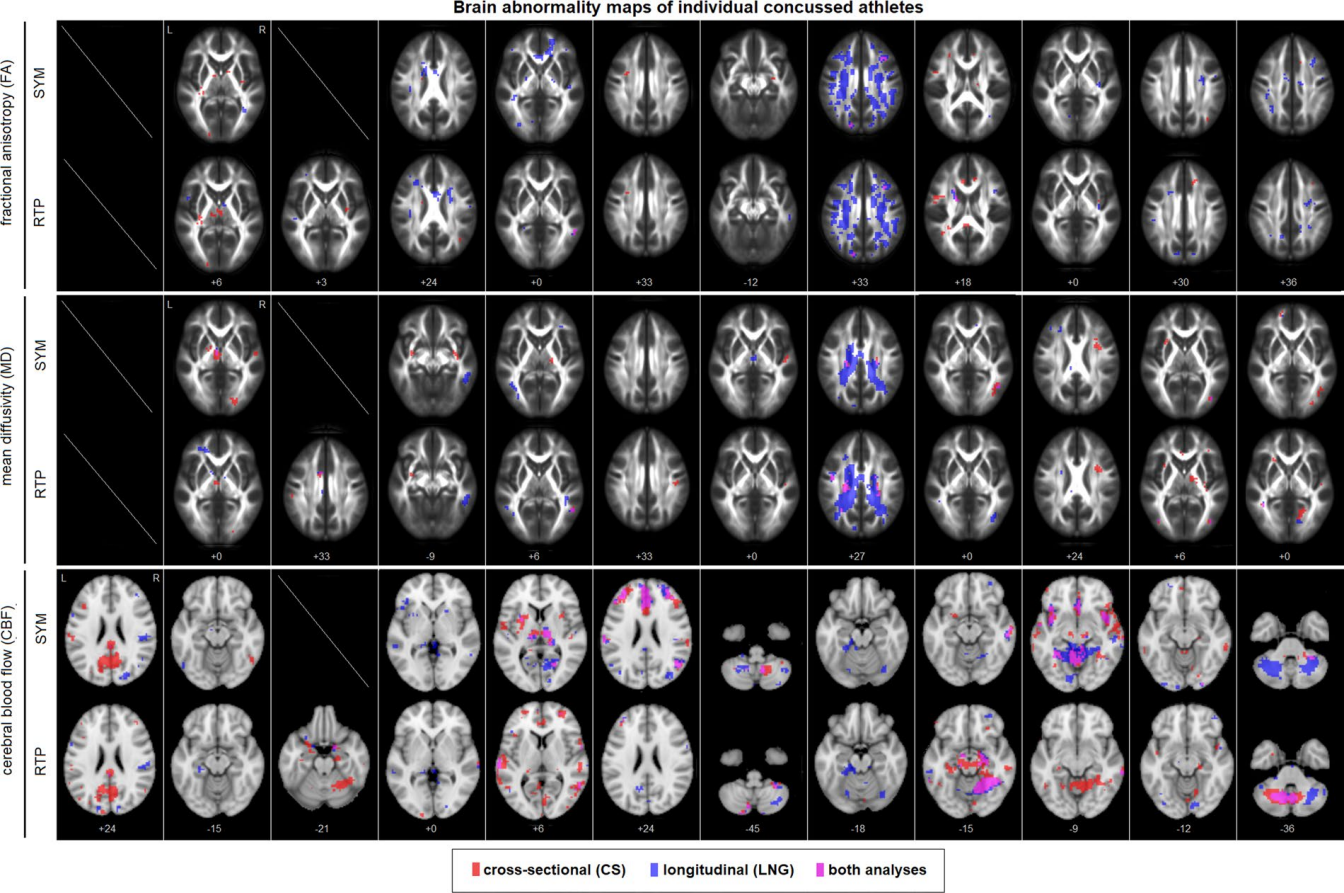
Areas of significant abnormality for individual concussed athletes, at the early symptomatic phase of injury (SYM) and medical clearance to return-to-play (RTP). A single representative axial slice is displayed per athlete, chosen to have greatest number of abnormal voxels, averaged over both analyses and time points (reported in Montreal Neurological Institute (MNI) space coordinates). No FA or MD results are shown for athlete #1 (SYM and RTP) due to missing baseline data, and no FA, MD or CBF results are shown for athlete #3 (SYM) as they could not be scanned during the symptomatic phase. Plots show variability of individual level abnormality maps, across subjects, analysis approaches and time points.

Brain abnormalities were identified for concussed athletes using both the CS and LNG analysis methods. For CS, a normative distribution was fit to athletic control baseline scan data at each voxel; for concussed athlete brain maps, significantly abnormal voxels were then identified at a False Discovery Rate (FDR) of 0.05. For LNG, a normative distribution was fit to athletic control brain changes (post-season – baseline) at each voxel; for concussed athlete brain maps of change (post-injury – baseline), significantly abnormal voxels were then identified at an FDR of 0.05. In both cases, the normative distributions were obtained using a robust kernel density estimator to minimize distributional assumptions. In Fig. 2, selected slices of the thresholded abnormality maps are shown for each concussed athlete, at both SYM and RTP time points. In general, the concussed athletes exhibited a high degree of variability in both the localization and spatial extent of brain abnormalities. Moreover, when comparing the CS (red) and LNG (blue) abnormality maps within subjects, there was often limited spatial overlap (purple) between the analyses.

**Figure 3:**
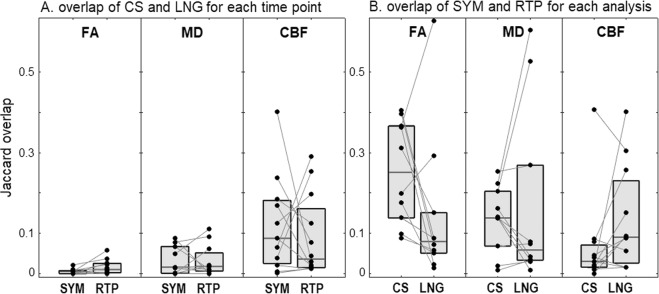
Fractional overlap of thresholded abnormality maps (False Discovery Rate = 0.05). This includes (**A**) comparison of cross-sectional (CS) and longitudinal (LNG) abnormality maps obtained at each time point, and (**B**) comparison of maps obtained at early symptomatic (SYM) and at return to play (RTP) time points obtained using both analysis approaches. Circles represent individual subject values and grey boxes denote the distribution quartiles. Relatively low spatial overlap is observed between CS and LNG models and between SYM and RTP time points, for all MRI parameters.

The similarity of CS and LNG thresholded abnormality maps was evaluated for each subject and post-concussion time point using the Jaccard index, which measures fractional overlap and ranges from 0 (no overlap) to 1 (perfect overlap). As shown in Fig. 3A, the analyses tended to identify distinct spatial patterns of brain abnormalities, as all MRI parameters had median overlap values that were below 0.10. In addition, no differences were seen in the overlap between analysis approaches for SYM and RTP time points (p ≥ 0.074 for all MRI parameters, paired Wilcoxon tests), therefore pooled statistics are reported. The overlap values were lowest for FA (0.007, [0.004, 0.015]), while MD had intermediate values (0.020, [0.011, 0.042]) and CBF had the highest values (0.120, [0.030, 0.156]).

The Jaccard index was also used to evaluate the similarity of thresholded abnormality maps identified at SYM and RTP time points, for each subject and analysis method. As depicted in Fig. 3B, the two time points identified substantially different spatial patterns of abnormalities, as all MRI parameters had median values below 0.20. No differences were seen in the overlap between time points for CS and LNG analyses (p ≥ 0.106 for all MRI parameters), therefore pooled statistics are reported in text. Overlap was highest for FA (0.212, [0.095, 0.246]), followed by MD (0.095, [0.071, 0.261]) and CBF had the lowest values (0.067, [0.033, 0.147]). For both CS and LNG analyses, the amount of spatial overlap between time points was not significantly correlated with the time interval between scans (|ρ| ≤ 0.293 and p ≥ 0.400 for all analyses).

Subsequent analyses compared the extent of abnormal voxels for CS and LNG analyses, at both SYM and RTP time points. Table 3 reports the percentage of significantly abnormal voxels for each MRI parameter, with the effects of analysis method and time point evaluated within a generalized linear mixed effects model. The LNG approach identified a higher percentage of abnormal voxels than CS for FA (*t*_(39)_ = 3.23; *p* = 0.002) and MD (*t*_(39)_ = 2.41; *p* = 0.021) but not for CBF (*t*_(43)_ = 0.70; *p* = 0.48), whereas there were no significant differences between SYM and RTP (*p* ≥ 0.335 for all MRI parameters). For LNG analyses, the percentage of abnormal voxels was not significantly correlated with time from baseline imaging (|ρ| < 0.360 and *p* ≥ 0.307 for all MRI parameters). Additional analyses of pre-injury baseline data using the CS approach also identified abnormal voxels prior to injury, for FA (0.43%, [0.17%, 1.71%]), MD (0.34%, [0.13%, 0.58%]) and CBF (1.26%, [0.70%, 4.79%]), with percentages that were not significantly different from acute injury (*p* ≥ 0.361 for all MRI parameters).

**Table 3:** The percentage of brain voxels that are identified as abnormal. Values are reported as median and [Q1, Q3] for both cross-sectional (CS) and longitudinal (LNG) analyses, at both early symptomatic (SYM) and return to play (RTP) time points.

	FA	MD	CBF
CS-SYM	0.30% [0.21, 0.68]%	0.49% [0.19, 1.26]%	2.48% [0.60, 5.98]%
CS-RTP	0.36% [0.27, 0.76]%	0.46% [0.29, 0.81]%	1.45% [0.69, 5.58]%
LNG-SYM	1.06% [0.25, 2.21]%	0.46% [0.24, 1.10]%	1.88% [1.32, 6.22]%
LNG-RTP	0.98% [0.52, 1.88]%	1.16% [0.31, 1.73]%	1.54% [1.06, 3.40]%

Table 4 reports the fraction of abnormal voxels that had “positive” abnormalities for each MRI parameter (i.e., values that were high relative to athletic controls; the remainder were low relative to athletic controls). There were no significant differences between CS and LNG analyses (*p* ≥ 0.095, for all MRI parameters) and FA showed no significant changes from SYM to RTP (*t*_(39)_ = −0.61; *p* = 0.547). Conversely, MD showed a significant longitudinal increase (*t*_(39)_ = 2.20; *p* = 0.034) and CBF showed a significant longitudinal decrease (*t*_(43)_ = −2.62; *p* = 0.012) in positive abnormalities. For LNG analyses, the fraction of positive abnormalities was not significantly correlated with time from baseline imaging (|ρ| < 0.450, p > 0.192). Additional analyses of the pre-injury baseline data using the CS approach found values intermediate between SYM and RTP for FA (0.316, [0.052, 0.876]) and CBF (0.627 [0.267, 0.782]), whereas MD had values lower than both SYM and RTP (0.546, [0.181, 0.713]), although not significantly different from acute injury (p ≥ 0.054 for all MRI parameters).

**Table 4:** The fraction of abnormal voxels that have “positive” abnormalities (i.e., values higher than controls; the remaining abnormal voxels are lower than controls). Values are reported as median and [Q1, Q3] for both cross-sectional (CS) and longitudinal (LNG) analyses, at both early symptomatic (SYM) and return to play (RTP) time points.

	FA	MD	CBF
CS-SYM	0.469 [0.075, 0.865]	0.689 [0.096, 0.882]	0.790 [0.450, 0.967]
CS-RTP	0.256 [0.050, 0.738]	0.900 [0.582, 1.00]	0.203 [0.053, 0.885]
LNG-SYM	0.630 [0.375, 0.947]	0.378 [0.121, 0.651]	0.862 [0.473, 0.984]
LNG-RTP	0.609 [0.371, 0.785]	0.696 [0.268, 0.914]	0.312 [0.100, 0.712]

### Neuroimaging data: spatial localization

**Figure 4:**
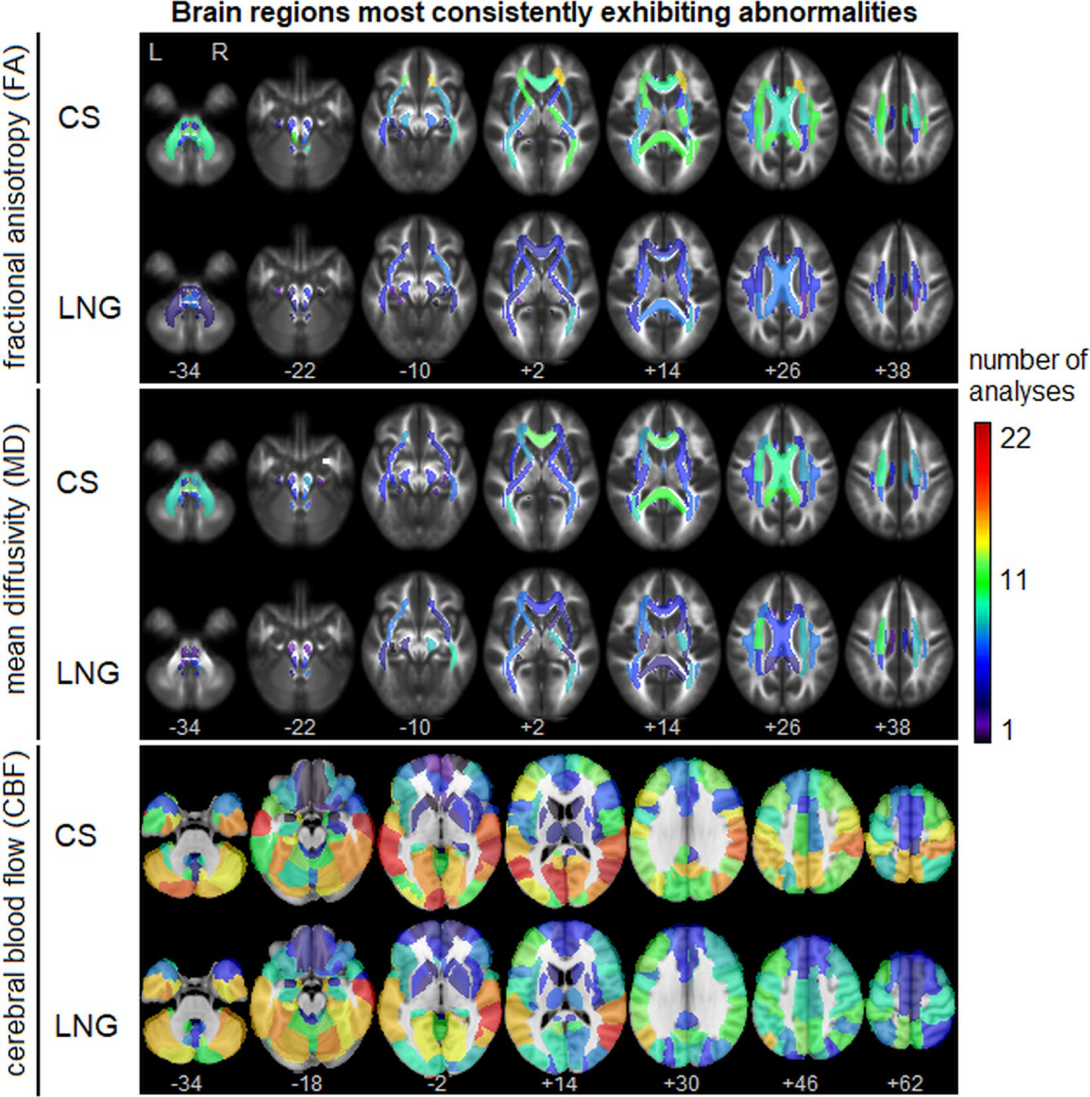
Heat maps showing brain regions most consistently exhibiting abnormalities for both cross-sectional (CS) and longitudinal (LNG) analyses. Maps depict the total number of analyses showing a significant abnormality at each brain region, summed over all participants and both imaging time points (both early symptomatic injury (SYM) and return to play (RTP)). Brain regions are segmented for FA and MD based on the John Hopkins University (JHU) white matter atlas and for CBF based on the automated anatomical labelling (AAL) atlas. Brain abnormalities are most prevalent in right posterior thalamic radiation and anterior corona radiata (FA); left superior corona radiata genu of the corpus callosum (MD); and bilateral middle temporal gyri (CBF).

Although this study focused on individual subject concussion-related abnormalities, supplemental analyses identified the brain regions most consistently exhibiting abnormalities. Figure 4 depicts the frequency that brain abnormalities are identified within each brain region, summed over all subjects and both post-concussion time points. For FA, abnormalities were most consistently seen across both analyses in the right posterior thalamic radiation, with 11 for CS (CS-SYM: 6, CS-RTP: 5) and 8 for LNG (LNG-SYM: 4, LNG-RTP: 4); along with the right anterior corona radiata, with 14 for CS (CS-SYM: 7, CS-RTP: 7) and 5 for LNG (LNG-SYM: 1, LNG-RTP: 4). For MD, abnormalities were most consistently in the left superior corona radiata, with 10 for CS (CS-SYM: 4, CS-RTP: 6) and 10 for LNG (LNG-SYM: 5, LNG-RTP: 5); along with the genu of the corpus callosum, with 12 for CS (CS-SYM: 5, CS-RTP: 7) and 5 for LNG (LNG-SYM: 2, LNG-RTP: 3). For CBF, frequencies of abnormalities were highest and were most consistently identified in the right middle temporal gyrus, with 21 for CS (CS-SYM: 10, CS-RTP: 11) and 19 for LNG (LNG-SYM: 8, LNG-RTP: 11); along with the left middle temporal gyrus, with 19 for CS (CS-SYM: 9, CS-RTP: 10) and 15 for LNG (LNG-SYM: 7, LNG-RTP: 8). The concordance of the CS and LNG frequency maps was also evaluated for each MRI parameter, by measuring the Spearman correlation between regional frequency values (along with the bootstrapped 95% confidence interval). Moderately high concordance was observed for FA (0.486, [0.113, 0.526]), lower concordance was seen for MD (0.363, [0.058, 0.449]) and relatively high concordance was seen for CBF (0.793, [0.568, 0.773]).

### Neuroimaging data: threshold effects

**Figure 5:**
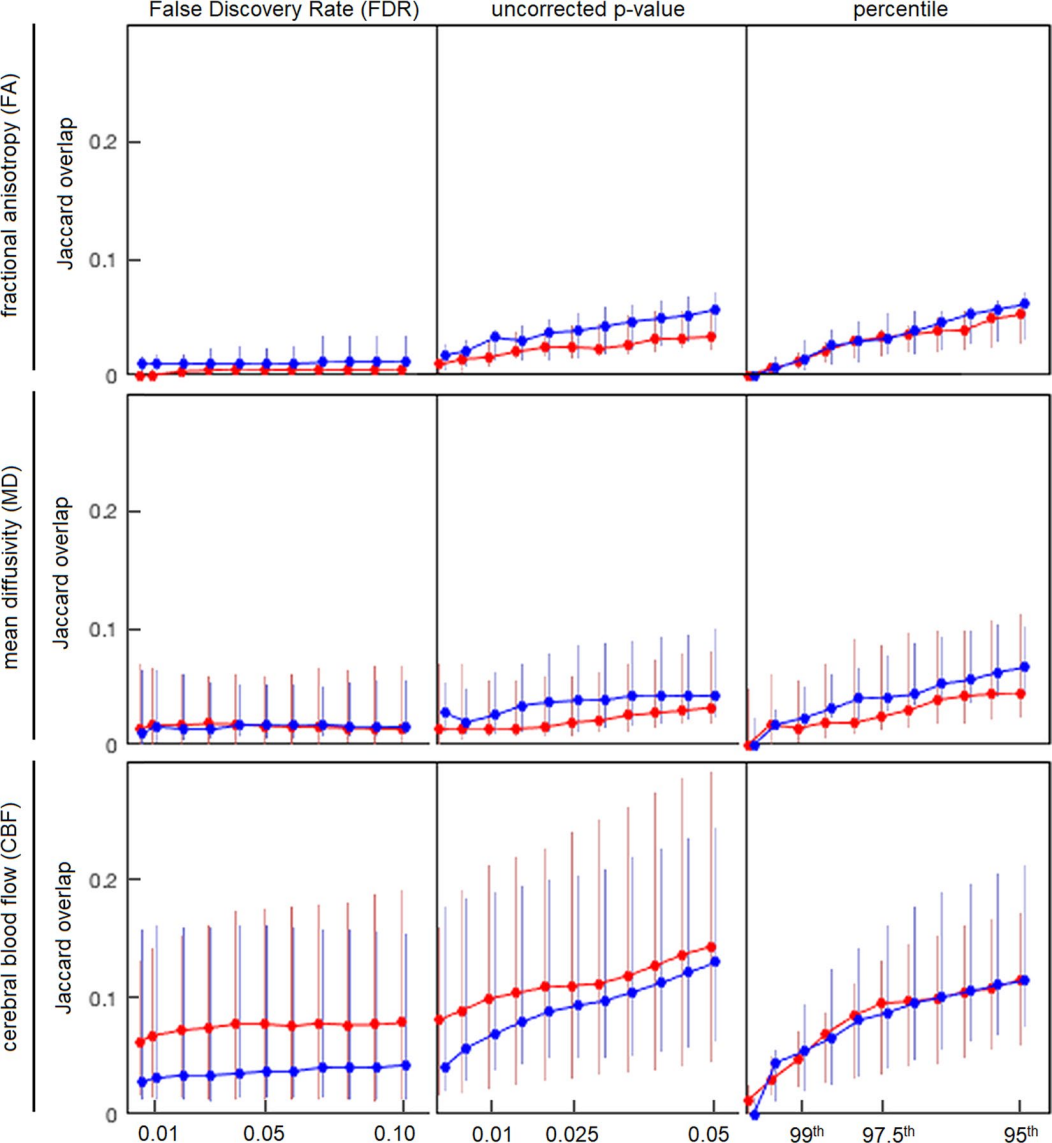
Jaccard overlap of thresholded cross-sectional (CS) and longitudinal (LNG) abnormality maps at both early symptomatic injury (SYM; red) and at return to play (RTP; blue), for different thresholding schemes. Solid lines denote median values, with interquartile error bars. This includes False Discovery Rate (FDR) thresholding, uncorrected p-values and a fixed percentile threshold. Low overlap is consistently observed between CS and LNG, for all thresholding approaches.

A second set of supplemental analyses determined whether the primary findings of this study (i.e., low overlap between CS and LNG methods) were dependent on the choice of threshold. Figure 5 plots the median overlap between CS and LNG, for both SYM (red) and RTP (curve) time points, under different thresholding schemes. The left column plotted results for a range of FDR thresholds, the middle column plotted results for a range of uncorrected p-value thresholds, and the right column plotted results for a range of percentile thresholds. Across all thresholding schemes, the overlap remained relatively low. In general, FDR results were unaffected by choice of threshold, whereas p-value and percentile methods produced increased overlap under more liberal thresholds, but the median fractional overlap did not exceed 0.15 for any of the studied MRI parameters.

## Discussion

In this multi-modal MRI study, concussion-related brain abnormalities were examined at the individual subject level, as this remains an understudied area of research. The focus of this study was on whether analyses of longitudinal change post-injury relative to pre-injury baseline (LNG) identified substantially different concussion effects than cross-sectional analyses of post-injury data (CS). This was evaluated for multiple different MRI measures (FA, MD and CBF), at the early symptomatic phase of injury (SYM) and following medical clearance to return to play (RTP). The primary study finding was that CS and LNG analyses identified distinct patterns of post-concussion brain abnormalities, indicating that the localization of concussion effects at the individual level is highly dependent on the choice of normative reference.

The primary findings were quantified in terms of Jaccard overlap between thresholded abnormality maps for CS and LNG analyses. Although individual subjects exhibited variable overlap values, the median for all MRI parameters was below 0.10. In addition, for DTI measures, the LNG analyses tended to identify a higher percentage of abnormal voxels compared to CS analyses. However, CS and LNG analyses were not significantly different in the amount of overlap between SYM and RTP time points, or the fraction of abnormal voxels with “positive” abnormalities. This suggests that the two approaches may have similar sensitivity for some aspects of concussion, and the differences are mainly in the specific brain regions identified. Comparing MRI parameters, CBF maps had the highest overlap between CS and LNG analyses, suggesting that it may be least influenced by baseline differences, which is consistent with FA and MD being intrinsic markers of neuroanatomy that exhibit significant, reliable differences between individuals^29^.

In interpreting the spatial overlap results, baseline imaging may provide a more robust reference, as it controls for sources of pre-injury brain variability, which include demographic factors of age, sex and concussion history^17–20^, along with more complex genetic contributions^30,31^. However, the LNG design also comes with some limitations, as the interval between scans for athletic controls includes more season play than for concussed athletes. In this time, there may be additional longitudinal changes driven by physical exertion^32^, exposure to sub-concussive impacts in contact/collision sports^33–35^ and other factors including differences in psychological stress^36,37^, which may influence estimates of normative variability. However, correlation analyses failed to find a significant association between time since baseline imaging and the extent or direction of brain abnormalities, suggesting a limited impact on the present study findings.

Other issues of demographic matching may affect both CS and LNG analyses; brain function and structure show systematic differences associated with age, sex, history of concussion and participation in contact/collision sports^17,33,35,38–40^, leading to potential biases (positive or negative) in the detection of brain abnormalities. However, this study evaluated overlap for a range of different thresholding approaches, which confirms that differences between CS and LNG analyses are not driven by differences in sensitivity. Potential biases in the LNG design may be quantified in future studies, by imaging athletic controls at multiple points throughout their competitive season and evaluating the effect of time on the detection of concussion-related abnormalities. Future work should also examine whether improved demographic matching of normative cohorts significantly affects the results of CS and LNG analyses.

This study also examined the evolution of brain abnormalities from SYM to RTP at the individual level. Limited spatial overlap was seen between time points, for both the CS and LNG analyses. Although subjects exhibited variable overlap values, the median for all MRI parameters was less than 0.20. This indicates extensive change in the spatial patterns of abnormalities seen at the individual level over the course of clinical recovery, consistent with existing literature showing longitudinal brain changes^16,41^. In addition, the DTI measures showed greater spatial overlap than CBF, indicating that concussion-related changes in white matter may be more spatially stable than CBF over time. Interestingly, the percentage of abnormal voxels did not decline significantly from SYM to RTP, suggesting that brain recovery may be incomplete at the time of RTP. These findings are consistent with group-level MRI studies reporting persistent effects at RTP for brain function and structure, both in this cohort^42^ and in others^41,43–45^.

In contrast, the direction of abnormality (positive or negative relative to uninjured athletes) showed significant effects of time, for both MD and CBF. MD tended to be low in symptomatic concussed athletes, which is consistent with prior literature^10,43,44^, although elevated diffusivity also been identified in some symptomatic athlete groups^12,46^. Interestingly, while the present study identified a longitudinal change from low to high MD, longitudinal group studies have typically reported the direction of effect to be unchanged or resolved over time^12,43,44^. The analysis of individual subject abnormality maps may therefore be able to track more complex, spatially heterogeneous changes in white matter diffusivity longitudinally. These findings may be contrasted with the lack of significant longitudinal effects for FA, suggesting that this DTI metric may be more variable in direction of change. For CBF, the values tended to be high at symptomatic injury but low at RTP. This is consistent with studies of young adults where elevated CBF has been reported at early injury, followed by a delayed decrease at or beyond the first week post-injury^14,47,48^.

Analyses of pre-injury baseline data using a CS approach also identified abnormalities in all MRI parameters prior to the concussion event of interest. This may reflect limitations of the CS approach, i.e., false positives due to brain variability unrelated to the most recent concussion, including prior history of concussion and exposure to subconcussive impacts. A similar LNG analysis, with multiple pre-injury scans, is required to determine whether such abnormalities are specific to the CS approach. This also suggests a method for refining brain map thresholds, i.e., using receiver operating curve techniques to optimally balance rates of true positives (post-concussion abnormalities) against false positives (pre-concussion abnormalities)^49^. However, pre-injury abnormalities may also of clinical interest, as potential early indicators of concussion risk. Studies have identified demographic and serum biomarkers risk factors in concussion^50,51^, but this has not been established for neuroimaging data to our knowledge. This suggests an intriguing area of future research and cautions against presuming pre-injury abnormalities to be uninformative false positives.

Although this study emphasized concussion effects at the individual level, supplemental analyses also identified the most consistently affected brain regions for the group. Analyses of the DTI parameters found the corona radiata and corpus callosum to be most consistently affected by concussion. This is consistent with prior models of brain injury biomechanics^52^ which found central white matter structures to be among the most vulnerable to injury. Moreover, it is aligned with a prior meta-analysis of DTI in mild TBI^53^, along with literature on sub-concussive impacts^33^, in which effects were most frequently seen in the corona radiata and corpus callosum. For CBF, the most reliably affected brain regions were the temporal lobes, which have been previously identified as vulnerable to primary impacts from concussion^54,55^, although interestingly the effects seem less prevalent frontally, which has been similarly identified as a vulnerable grey matter region^55,56^. Nevertheless, the analyses of the most affected brain regions are generally consistent with the existing conceptualization of brain injury biomechanics and associated pathophysiology.

This study focused on the detection of statistically significant brain abnormalities among concussed athletes, relative to a normative cohort. While the main objective of concussion neuroimaging research is the quantification of changes in brain physiology, threshold-based analysis plays a key role in current practise. A statistical criterion is needed to determine whether post-concussion brain physiology is different from uninjured brains in a way that exceeds normal variability. Moreover, thresholding is widely used to simplify and summarize high-dimensional brain images, providing regions of interest in which we can further quantify the changes in MRI parameters. This study focused on FDR-based thresholding of univariate p-value maps, but also assessed thresholding based on uncorrected p-values and percentiles. This is by no means exhaustive and other approaches may be used, including alternative univariate thresholds (e.g., based on cluster size), multivariate tests of abnormality (e.g., Mahalanobis distance) and machine learning methods (e.g., one-class classifiers). An important area of future investigation will be the evaluation and comparison of alternative procedures for detecting concussion-related abnormalities.

There is growing recognition of the importance of considering the individual when using MRI to evaluate concussion. Substantial inter-individual differences have been seen in the patterns of concussion-related pathophysiology^23,24,27^ potentially stemming from injury biomechanics^52^, but also likely confounded by variations in pre-injury brain physiology^17–20^. Although the present study was based on a limited sample size, the findings, in combination with emerging literature^26,27,57^, underscore the importance of research that includes baseline pre-injury data, to better characterize the neurobiology of concussion and validate existing group-level cross-sectional studies. The primary study finding, of distinct spatial patterns of concussion-related abnormalities for baseline and cross-sectional analyses, suggest that the use of pre-injury baseline data may be most critical in the identification of most sensitive brain regions for biomarkers and/or interventions^9^. Although it may be infeasible to collect pre-injury MRI data in many non-athlete cohorts, these findings may help to determine methods for improving future cross-sectional analyses, such as improved selection procedures for matched controls.

## Materials and Methods

### Study participants

A total of 123 athletes were imaged at the start of their competitive season, drawn from volleyball, hockey, soccer, football, rugby, basketball, lacrosse and water polo. From this group, 12 athletes (10% of the cohort) were re-imaged after sustaining a concussion, (1) at the early symptomatic phase of injury and at time of RTP. In addition, 44 athletic controls, drawn from the remaining athletes that did not sustain a concussion, were re-imaged at the end of their competitive season. For concussed athletes, diagnosis was determined by a staff physician following events where athletes sustained direct or indirect contact to the head with the presence of signs and/or symptoms as per the Concussion in Sport Group guidelines^58^ and RTP was determined based on symptom resolution following a graded exertional protocol^3^.

All athletes completed pre-season Sport Concussion Assessment Tool (SCAT)^59,60^ assessments. Furthermore, all athletes with concussion completed SCAT assessments as part of initial concussion assessment and at RTP. This study was carried out in accordance with the recommendations of the Canadian Tri-Council Policy Statement 2 (TCPS2) and with approval of the research ethics boards at the University of Toronto and St. Michael’s Hospital, with participants giving free and written informed consent in accordance with the Declaration of Helsinki.

### Magnetic resonance imaging

Athletes were imaged using a 3 Tesla MRI system (Magnetom Skyra) with a standard multi-channel head coil. Structural imaging included the following protocol: three-dimensional T1-weighted Magnetization Prepared Rapid Acquisition Gradient Echo imaging (MPRAGE: inversion time (TI)/echo time (TE)/repetition time (TR) = 1090/3.55/2300 ms, flip angle (FA) = 8°, 192 sagittal slices with field of view (FOV) = 240 × 240 mm, 256 × 256 pixel matrix, 0.9 mm slice thickness, 0.9 × 0.9 mm in-plane resolution, with bandwidth (BW) = 200 Hertz per pixel (Hz/px)), fluid attenuated inversion recovery imaging (FLAIR: TI/TE/TR = 1800/387/5000 ms, 160 sagittal slices with FOV = 230 × 230 mm, 512 × 512 matrix, 0.9 mm slice thickness, 0.4 × 0.4 mm in-plane resolution, BW = 751 Hz/px) and susceptibility-weighted imaging (SWI: TE/TR = 20/28 ms, FA = 15°, 112 axial slices with FOV = 193 × 220 mm, 336 × 384 matrix, 1.2 mm slice thickness, 0.6 × 0.6 mm in-plane resolution, BW = 120 Hz/px). To rule out potential structural abnormalities, the MPRAGE, FLAIR and SWI scans were reviewed in a 2-step procedure, with initial inspection by an MRI technologist during the imaging session and later review by a neuroradiologist with clinical reporting if abnormalities were identified. No abnormalities (white matter hyper-intensities, contusions, micro-hemorrhage, or statistical outliers) were found for the concussed athletes and controls in this study.

#### Diffusion tensor imaging

A diffusion weighted imaging protocol was performed (66 axial slices with FOV = 240 × 240 mm, 120 × 120 matrix, 2.0 mm slice thickness, 2.0 × 2.0 in-plane resolution, BW = 1736 Hz/Px), consisting of 30 diffusion-weighting directions (TE/TR = 83/7800 ms, b = 700 s/mm^2^, with 9 b0 scans). This sequence was from an earlier study in which multiple different b-values were acquired^61,62^. Due to time limitations, only the b = 700 was collected for participants in this study, as it had greatest sensitivity to concussion-related brain changes^61^. The DTI data were processed using utilities from the fMRIB Software Library (FSL; https://fsl.fmrib.ox.ac.uk/fsl/fslwiki) and software developed in-house. The FSL *eddy* protocol was used to perform simultaneous correction of eddy currents and rigid-body head motion, FSL *bet* was used to mask out non-brain voxels, and FSL *dtifit* was used to calculate voxel-wise FA and MD. Co-registration of brain maps was based on the FSL FDT protocol: (1) masked subject FA maps were eroded by 1 voxel width at brain edges, and co-registered to the FMRIB58 template via affine transform using FSL *flirt*; (2) a symmetric, study-specific template was computed by averaging transformed FA maps, then re-averaging with flipped left/right orientations; (3) the average template was used as a reference and non-linear registration of FA maps performed using FSL *fnirt*, which were used to update the study-specific template; (4) the FA maps were registered to the new template via *fnirt* and the mean template was updated. During the final registration step, images were resampled to 3 × 3 × 3 mm resolution, and prior to analysis all images were convolved with an 8 mm FWHM 3D Gaussian smoothing kernel to minimize the effects of local variation in white matter structure. All analyses were performed within a mask of regions with a mean FA > 0.25, to restrict analyses to white matter tracts.

#### Arterial spin labelling

2D pulsed arterial spin labelling (ASL) was acquired using the PICORE QUIPSS II sequence (TE/TR = 12/2500 ms, TI1/TI1s/TI2 = 700/1600/1800 ms, FA = 90°, 14 oblique-axial slices with FOV = 256 × 256 mm, 64 × 64 matrix, 8.0 mm slice thickness with 2.0 mm gap, 4.0 × 4.0 mm in-plane resolution, BW = 2368 Hz/px). A single M_0_ calibration scan and a series of forty-five tag-control image pairs were acquired. Data were processed and analyzed via a combination of Analysis of Functional NeuroImages (AFNI; https://afni.nimh.nih.gov), FSL and software developed in-house. Rigid-body motion correction of tag-control scans was performed using AFNI *3dvolreg*, aligning the images to the M_0_ scan. We then performed filtering of outlier tag-control pairs using the protocol of^63^, followed by spatial smoothing with AFNI *3dmerge*, using a 6 mm isotropic 3D Gaussian kernel. Voxel-wise CBF estimates were calculated in units of mL/100 g/min based on the mean difference over all tag-control pairs, using the kinetic modelling parameters previously applied in^14^. Co-registration of CBF images was obtained by (1) the rigid-body transform of each participant’s mean functional volume to their T1 anatomical image via FSL *flirt*, and (2) the 12-parameter affine transformation of their T1 image to the MNI152 template using *flirt*. These transformation matrices were concatenated and the net transform applied to all functional data, resampled at 3 × 3 × 3 mm resolution. Analyses were restricted to a mask of grey matter regions, where mean CBF for controls was >20 mL/100 g/min.

### Neuroimaging data: normative controls

The normative data were displayed for reference, including maps of the voxel-wise medians and interquartile ranges (Fig. 1). As summative measures of inter-individual variation, the coefficient of variation *CoV* = *std*(*X*)/|*mean*(*X*)| was also calculated at each voxel and reported as the median and [Q1, Q3] over all voxels. In addition, the median percent signal change was calculated longitudinally in the control data *CHG* = 100 ∗ ([*post*−*season*] − [*baseline*])/[*baseline*] and also reported as the median [Q1, Q3] over all voxels.

### Neuroimaging data: concussed athletes

Brain abnormalities of individual concussed athletes were identified for FA, MD and CBF brain maps. Voxel-wise normative distributions were obtained for athletic controls via kernel density estimation (KDE), which were then used to identify significantly abnormal voxel values for concussed athletes. The KDE approach provides a flexible non-parametric technique for identifying outlying values with minimal assumptions about the underlying dataset. This approach was chosen because, while data on average were well approximated by a normal distribution, a non-trivial fraction of voxels exhibited significant deviations from normality (FA-CS: 5.3%, FA- LNG: 21.2%, MD-CS: 18.3%, MD- LNG: 16.7%, CBF-CS: 10.6%, CBF- LNG: 13.8%, voxel-wise Shapiro-Wilk tests, at a threshold of FDR = 0.05). Given the relatively sparse nature of abnormalities and low overlap values, mismodelling within these voxels may therefore have a substantial impact, particularly since the extent of non-normality varies by MRI parameter and analysis method.

The KDE was applied to control datapoints *X*_*ctl*(*n*)_ (*n* = 1…*N*_ctl_) using a Gaussian kernel basis. The kernel bandwidth *h* was obtained by performing leave-out-out cross-validation across a range of kernel sizes (0.01 to 10 times the sample standard deviation) and identifying the value minimizing mean squared error: $$CV(h)=\int {\hat{f}}_{b}^{2}(x)dx$$$$-\frac{2}{N}\mathop{\sum }\limits_{n=1}^{Nctl}\,{\hat{f}}_{b,n}({X}_{ctl(n)})$$^64^. The Gaussian kernel weights were subsequently determined for control datapoints using the robust KDE approach^65^ with code adapted from [web.eecs.umich.edu/~cscott/code.html#rkde] and a Hampel loss function. The KDE distributions were then evaluated for concussed data points *X*_*conc*(*n*)_ (*n*=1…*N*_conc_), to obtain cumulative probability values *P*_*conc*(*n*)_, which were converted into 2-tailed cumulative probabilities $${P}_{conc(n)}^{^{\prime} }=2\,\ast \,{\rm{\min }}([{P}_{conc(n)},\,1-{P}_{conc(n)}])$$, reflecting the probability of a value as extreme or larger originating from the control distribution.

This approach identified voxel-wise abnormalities in concussed athlete brain maps, using two different analysis approaches. For cross-sectional analysis (CS), the normative data were athletic control baseline scan values; for each concussed athlete scan, probabilities were calculated for both SYM and RTP time points. For longitudinal analysis (LNG), the normative data were athletic control changes in scan values (post-season – baseline); for each concussed athlete scan, probabilities were calculated on the changes (post-injury – baseline), for both SYM and RTP time points. This produced probability maps for each concussed athlete, MRI parameter and post-injury time point. The maps were then thresholded to produce binary maps of brain abnormalities, at a False Discovery Rate (FDR) of 0.05, with an additional minimum cluster-size threshold of 3 imposed, to remove singleton clusters. The thresholded abnormality maps were displayed for all concussed athletes and time points, with CS and LNG abnormality maps shown with overlapping regions. A representative axial slice was chosen per athlete, by ranking axial slices of each of the 4 abnormality maps (SYM/RTP x CS/ LNG) by total number of abnormal voxels, then selecting the slice of highest mean rank.

The similarity of CS and LNG abnormality maps was evaluated using the Jaccard index, which quantifies fractional overlap. For binary brain maps ***X*** and ***Y***, this is defined as:

$$J({\boldsymbol{X}},{\boldsymbol{Y}})=\,{\boldsymbol{X}}{\cap }^{}{\boldsymbol{Y}}/{\boldsymbol{X}}{\cup }^{}{\boldsymbol{Y}}$$

This value was calculated for each subject and time point, and differences in overlap between SYM and RTP were evaluated using nonparametric paired-measures Wilcoxon tests. In addition, longitudinal changes in abnormalities were assessed by measuring the Jaccard index for subject SYM and RTP maps, with a lower overlap indicating greater longitudinal change in spatial pattern. This value was calculated for each subject and analysis method, and differences in overlap between CS and LNG were evaluated using nonparametric paired-measures Wilcoxon tests.

The abnormality maps were also characterized in terms of spatial extent and direction of effect. The spatial extent was evaluated by calculating the fraction of voxels showing significant abnormality, for each subject, analysis method and time point. In addition, the direction of effect was evaluated by determining, for abnormal voxels, whether they were “positive” (i.e., in the upper tail *P*_*conc*(*n*)_ ≥ 0.975; high relative to athletic controls) or “negative“ (i.e., in the lower tail *P*_*conc*(*n*)_ ≤ 0.025; low relative to athletic controls). The fraction of abnormal voxels that were positive was then calculated for each subject, analysis method and time point. For both measures, the effects of analysis method and time point were evaluated in a generalized linear mixed effects model (GLMM), with analysis model and time as fixed effects, and a random-effects intercept for each subject. To account for strictly positive response variables, a Gamma distribution was modeled with log link function and with maximum pseudo-likelihood fitting using the Matlab R2017b *fitglme* package (The MathWorks, Natick MA).

### Neuroimaging data: spatial localization

To summarize brain regions most consistently identified as abnormal, the following steps were performed: anatomical subdivisions of the brain were defined, using the John Hopkins University (JHU) white matter atlas^66^ for FA and MD and the automated anatomical labelling (AAL) grey matter atlas^67^ for CBF. Each abnormality map was then parcellated into spatially contiguous clusters. For each cluster, all overlapping anatomical regions were identified; if no regions directly overlapped, the template region was identified whose center of mass had the shortest Euclidean distance from the cluster center of mass. For each anatomical region, the number of abnormality maps having at least one cluster assigned to the region was then calculated. This was used to produce a frequency map, with higher numbers indicating greater frequency of abnormality.

### Neuroimaging data: threshold effects

To verify that the main findings of this study generalized beyond the fixed FDR = 0.05 threshold, overlap between CS and LNG maps was also calculated under different thresholding schemes. This included a range of different FDR thresholds (0.005 to 0.10), a range of nominal uncorrected p-value thresholds (0.005 to 0.05), and a range of percentile thresholds (99.9th to 95th). In each case, the median and [Q1, Q3] of the overlap values was plotted.

## Data Availability

The datasets generated during and/or analysed during the current study are available from the corresponding author on reasonable request.
